# Insulin Signaling as a Key Moderator in Myotonic Dystrophy Type 1

**DOI:** 10.3389/fneur.2019.01229

**Published:** 2019-11-26

**Authors:** Sylvia Nieuwenhuis, Kees Okkersen, Joanna Widomska, Paul Blom, Peter A. C. 't Hoen, Baziel van Engelen, Jeffrey C. Glennon

**Affiliations:** ^1^Department of Cognitive Neuroscience, Donders Institute for Brain Cognition and Behaviour, Radboud University Medical Centre, Nijmegen, Netherlands; ^2^Department of Neurology, Donders Institute for Brain Cognition and Behaviour, Radboud University Medical Centre, Nijmegen, Netherlands; ^3^VDL Enabling Technologies Group B.V., Eindhoven, Netherlands; ^4^Centre for Molecular and Biomolecular Informatics, Radboud Institute for Molecular Life Sciences, Radboud University Medical Centre, Nijmegen, Netherlands

**Keywords:** insulin, myotonic dystrophy (DM1), insulin-like growth factor 1 (IGF1), metformin, diabetes type 2, insulin resistance, obsessive–compulsive disorder, behavioral flexibility

## Abstract

Myotonic dystrophy type 1 (DM1) is an autosomal dominant genetic disease characterized by multi-system involvement. Affected organ system includes skeletal muscle, heart, gastro-intestinal system and the brain. In this review, we evaluate the evidence for alterations in insulin signaling and their relation to clinical DM1 features. We start by summarizing the molecular pathophysiology of DM1. Next, an overview of normal insulin signaling physiology is given, and evidence for alterations herein in DM1 is presented. Clinically, evidence for involvement of insulin signaling pathways in DM1 is based on the increased incidence of insulin resistance seen in clinical practice and recent trial evidence of beneficial effects of metformin on muscle function. Indirectly, further support may be derived from certain CNS derived symptoms characteristic of DM1, such as obsessive-compulsive behavior features, for which links with altered insulin signaling has been demonstrated in other diseases. At the basic scientific level, several pathophysiological mechanisms that operate in DM1 may compromise normal insulin signaling physiology. The evidence presented here reflects the importance of insulin signaling in relation to clinical features of DM1 and justifies further basic scientific and clinical, therapeutically oriented research.

## Introduction

Myotonic dystrophy type 1 (DM1) is an autosomal dominant disease caused by a trinucleotide (i.e., CTG) repeat expansion in the 3′ untranslated region of the dystrophia myotonica protein kinase (i.e., *DMPK)* gene on chromosome 19q13.3. Clinically, DM1 is an extremely variable, multi-organ disease. In the last decades, both clinical and scientific studies that will be reviewed here, have hinted the presence of alterations in insulin and insulin-related signaling in DM1. These alterations may provide an important pathophysiological link between the genetic defect in DM1 and some of its pleiotropic downstream effects. Thus, the purpose of this review is to provide an overview of evidence, obtained from basic and clinical studies, regarding alteration of insulin signaling in DM1. We shortly recapitulate DM1 pathophysiology, after which an overview of insulin signaling physiology is provided. We then present the clinical and molecular evidence for insulin signaling involvement in DM1 and end with a summary and suggestions for future research.

## Molecular Pathophysiology of DM1

The molecular cascades through which the expanded CTG repeat sequence leads to clinical DM1 features is partially understood. One key player is the Muscleblind-like 1 protein (MBNL1), which *in vivo* studies demonstrate to be associated with muscle impairments and cataracts. The leading idea is that *DMPK* CUG expansion RNA, reaches such levels to sequester and compromise MBNL1 cellular functions. This is caused by squelching or trapping of the *DMPK* repeat resulting in the formation of ribonuclear foci (1, 2) that sequester and disrupt activities of RNA binding proteins from the MBNL and CUG-BP Elav-like family (CELF) families. DM1 can be seen as an RNA-toxicity disease, in which the nuclear accumulation of aberrant *DMPK* mRNA transcripts harboring the CTG repeat expansion, lead to a secondary spliceopathy. This results in abnormal processing of many gene products (3). These pleiotropic downstream effects may partially explain the clinical phenotypic variability that is a hallmark of DM1. The RNA toxicity is the consequence of microsatellite repeat expansions including RAN translation and CELF1 up-regulation but these are in addition to the primary mechanisms, such as MBNL loss of function and haplo-insufficiency of the normal DMPK gene product (4–6). With regards to the latter, the normal product of the *DMPK* gene is a tail-anchored protein kinase which is present in cellular membranes (7). The protein is expressed in skeletal, smooth and heart muscle, as well as in central nervous system, but not in liver or adipocytes (8, 9).

Although the relative contributions of different pathophysiological cascades through which the CTG repeat expansion leads to the clinical DM1 phenotypes remain unknown, important clinical features are dependent on the characteristics of the CTG repeat expansion (10, 11). A longer CTG repeat expansion is associated with an earlier clinical age of onset of disease, and with increased disease severity (12). The increase in CTG repeat length from generation to generation as a consequence of germline repeat instability explains the clinical phenomenon of anticipation: more severe, earlier-onset disease in subsequent generations (13). Finally, within an individual, the increase in the length of the CTG repeat expansion during life (i.e., somatic repeat instability) in tissues is dependent upon the CTG repeat length at birth (14).

## Physiology of Insulin Signaling

The beta cells in the pancreatic Islets of Langerhans release insulin to maintain glucose homeostasis in response to elevated blood glucose levels. Insulin, a metabolic hormone, regulates the uptake of glucose into adipose tissue, muscle, liver, brain (15–17), and vasculature (18) but also has key functions in lipid metabolism and protein synthesis (19–21). Lipid metabolism is a dynamic biological process, whereby insulin is involved in the reduction of hepatic gluconeogenesis process by lipolysis inhibition and hepatic acetyl-CoA (acetyl coenzyme A) (19). Insulin is associated with the regulation of muscle protein synthesis, whereby decreased insulin sensitivity results in reduced muscle mass (20, 21). A key action of insulin is to act to regulate glucose tone. Elevated glucose levels in the blood following food intake, are detected by the beta cells in the pancreatic Islets of Langerhans which stimulate insulin release.

**Figure 1:**
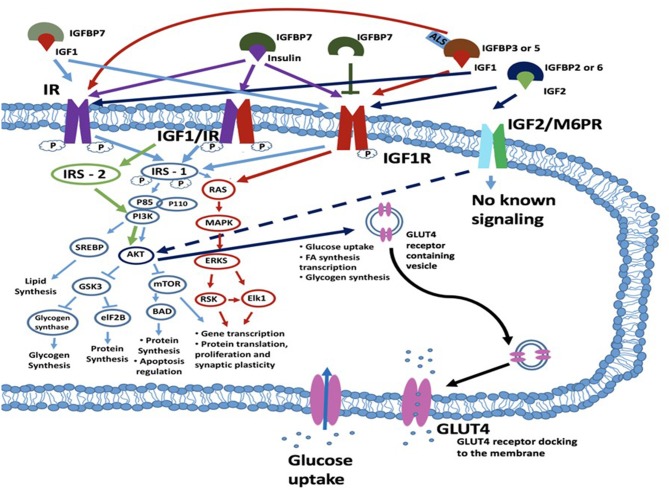
Schematic diagram of a cell with insulin/IGF signaling cascades depicted. Insulin may exert its effects through one of three transmembrane kinase receptors: IR, IGF1R or the IGFR/IR hybrid receptor. The IGF2R/M6P receptor has an unknown signaling potential. IGF1 can act alone or complexed with IGFBP3 or IGFBP5 and ALS and interacts with IGF1R and IR. IGF2 when bound to IGFBP2 or IGFBP6 interacts with the IGF2R (M6PR), IR or IGF1R. Insulin signaling is also regulated by IGFBP7, which can bind both IGF1 and insulin and bind with their receptors. IGFBP7 can also bind to the IGF1R which leads to the blockade of the IRS1 intrinsic pathway. Following ligand-receptor interaction, signal transduction to the cytosol is via the kinase domains of the IR, IGF1/IR and IGF1R receptors. The main targets for phosphorylation are the IRSs, which further transduce the signals through divergent messenger cascades, eventually leading to the various metabolic effects of insulin and IGFs. These effects can be metabolic or mitogenic and include lipid synthesis via PI3K/SREBP pathway, glycogen synthesis and protein synthesis and apoptosis regulation via the PI3K/AKT/GSK3/eIF2B pathway; and transcription and translation, cellular proliferation and synaptic plasticity via the RAS/MAPK/ERKS/RSK/Elk1 pathway. For an excellent review and more detail, see (23). IGF2 binds to the IGF2R (also known as the M6P receptor) and lacks intrinsic kinase activity but has been reported to regulate AKT activity. AKT acts to regulate multiple downstream targets including GSK3 and MTOR but is also involved in glucose uptake and the expression of GLUT4 which is a key player in glucose uptake. ALS, acid-labile subunit glycoprotein; eIF2B, eukaryotic initiation factor 2B; GSK3β, glycogen synthase kinase 3 beta; IGF1R, insulin growth factor receptor; IGFBP, insulin growth factor binding protein; IR, insulin receptor; IRS1, insulin receptor substrate-1; MAPK, mitogen-activated protein kinase; mTOR, mammalian target of rapamycin; RSK, ribosomal S6 kinase.

Insulin is produced by the cleavage of proinsulin and exerts its biological function through interaction with insulin binding receptors. These include the insulin receptor (IR), and the insulin growth factor 1 receptor (IGF1R) (22) (Figure 1). The IR belongs to the superfamily of transmembrane receptor tyrosine kinases. It is encoded by the insulin receptor (INSR) gene on chromosome 19p13.2. Alternative splicing of the INSR pre-mRNA leads to formation of two IR isoforms: IR-A and IR-B. These isoforms differ in that IR-A has a higher binding affinity for insulin, faster internalization and recycling time compared to IR-B. In contrast, IR-A has a lower signaling capacity and 2-fold lower tyrosine kinase activity compared to IR-B (24). Besides insulin, ligands structurally similar to insulin may also activate the insulin receptor, such as insulin growth factors. Equally, insulin may exert downstream effects through interaction with insulin growth factor receptors (25, 26). Binding to the insulin IR-A and IR-B receptors results in the activation of secondary cascades which interact with the insulin response element 1 (IRS1). This can interact with the RAS/mitogen-activated protein kinase (MAPK) cascade which regulates extracellular-signal-regulated kinase signaling (ERKS) (IRS1, RAS, MAPK, and ERKS are shown in Figure 1 circled in red). With consequent effects on gene and protein proliferation, and protein translation. In addition, IRS-1 can also regulate through phosphoinositide 3-kinases (PI3K) and Protein kinase B (PKB), also known as AKT (AKT)-related signaling which regulates protein production and apoptosis via mitogen-activated protein kinase (mTOR) on one side and glycogen synthesis via glycogen synthase kinase 3 beta (GSK-3β) on the other side [PI3K, AKT, mTOR, and GSK3β are illustrated in Figure 1 (blue circles)]. Activation of the IR or hybrid IGF1/IR receptor via IRS1-PI3K signaling can also regulate an additional mechanism, sterol regulatory element-binding proteins (SREBP) which regulates lipid synthesis. The major target tissues of insulin are liver, adipose tissue and skeletal muscle, but IRs also are expressed in blood cells, fibroblasts, brain and internal organs, such as lungs and kidneys (23).

Insulin growth factors (IGFs) are metabolic hormones that are structural homologs of insulin, and share common signaling pathways (25, 26). However, they also have key differences in functionality to insulin in part due to different affinities of insulin, IGF1 and IGF2 for the same targets. IGF1 and IGF2 are predominantly produced in the liver, but also in pancreas, intestine, kidney, brain, adipose tissue and muscle (16, 18, 27). These hormones mostly act in an autocrine or paracrine fashion and are produced locally (16, 27, 28). In addition, circulating IGF1 can also induce systemic effects. IGF1 is involved in the regulation of cellular and tissue growth by promoting anabolic action after birth throughout life. Several studies have demonstrated IGF1 and its receptor IGF1R mRNA synthesis in brain regions, such as olfactory bulb, cerebellum, and hippocampus (24). IGF1 promotes neurogenesis and has neuroprotective properties against cellular damage (29). IGF1 signaling plays a pivotal role in brain aging which is associated with a lowering of serum IGF1 levels (30). IGF1 from the circulation can cross the blood-brain barrier and potentially protect against neuronal apoptosis (16, 29). The lowering of serum IGF1 levels with age likely reduces the protective effects on brain and leaves the brain more vulnerable to insults. In muscle, IGF1 is associated with myoblast proliferation, myocyte synthesis and muscle repair. IGF1 administration in high levels for a long period results in muscle hypertrophy in mdx mice (31). IGFs production is closely regulated by the pituitary-derived growth hormone (GH) (30, 32). IGF2 has various known functions, such as an important role in hematopoiesis, inflammation and learning and memory (33–36).

The “natural” receptors for the IGFs through which IGF1 and IGF2 exert their effects are the insulin growth factor receptors (IGFRs): IGF1R and IGF2R. These receptors are located on the cellular membranes, concurrently expressed in the same tissues as IGF1 and IGF2, respectively. The targets of insulin and IGFs are not mutually exclusive and the IGF1R and IR may form a hybrid receptor complex that is responsive to both insulin and IGF1 binding (see Figure 1) (37–39). For successful interaction with their receptors, IGF1 and IGF2 require the presence of IGF binding proteins (IGFBP) and, in case of IGF1, acid-labile subunit protein (ALS, see below) (16, 18, 28, 40–42). These hybrid receptors are expressed in the human placenta and musculature (26, 43). IGF1 mainly exerts its various effects in multiple different tissues through the IGF1 and IGF1/IR hybrid receptor (42). IGF2 acts via binding to the IGF2 receptor which is also known as the mannose-6 phosphate (M6P) receptor (24, 28). For the IGF1R, downstream signaling mechanism have been elucidated and include activation of IRS1 and RAS signaling, comparable to IR activation. For IGF2R downstream mechanisms connected to the IGF2/M6P receptor remain unclear (44). Thus far, it has been established that this is independent of a tyrosine kinase-dependent mechanism but does involve ERK/GSK3β signaling. The IGF2/M6P receptor also functionally acts to clear IGF2 from the circulation. This is performed by transporting IGF2 ligands via the lysosomal system for degradation. As such the IGF2/M6P receptor acts to control the amount of circulating IGF2 available for interaction with other receptor types, such as the IGF1/IR receptors (44).

Insulin growth factor binding proteins are a group of 7 proteins that bind with high affinity to IGF1, IGF2, and insulin and modulate their coupling to the IGF and IR receptors (16, 18, 28). IGFBP1-6, are synthesized in brain and liver (24). These act to regulate IGF transmission and also play roles in myelination, synaptic plasticity and apoptosis in the brain (16, 29, 45–47). More generally, IGFBPs 1–6 are involved in growth, metabolism, cancer and immune regulation. In contrast to other IGFBPs, IGFBP7 is predominantly expressed in the gastrointestinal tract, lung, and prostate (48) but is also present in brain. It has a 500-fold higher binding affinity for free serum insulin than IGF1 and can therefore regulate insulin tone and action at its receptor (48, 49). The higher binding affinity of IGFBP7 to the IGF1 receptor leads to the blockade of the IRS1 intrinsic pathway which impairs activation of protein synthesis, cell proliferation and cell growth survival and apoptosis (50). While insulin resistance is also present in DM1 patients (51, 52), an upregulation of IGFBP7 remains to be observed. Besides the IGFBPs, IGF1 can also bind to the ALS glycoprotein which is produced in the liver and serum (41). The main function of ALS is to extend the half-life of IGF-IGFBP complexes in the circulation to act on the IGF1R and IR (41, 42). The ability of the IGFBPs 1–7 and ALS to modulate IGF and insulin action may provide a route toward the modulation of insulin and IGF signaling in DM1. It should be stated however that this is one of many mechanisms available to regulate IGF1/insulin action.

## Clinical Evidence of Insulin Signaling Involvement in DM1

### Insulin and Insulin Resistance in DM1

In DM1, alterations in insulin metabolism are well-recognized features of the disease (53–55). DM1-related changes in insulin signaling have been reported in ~30 clinical studies over the last six decades (summarized in Table 1). The majority of studies found increased plasma insulin levels (i.e., hyperinsulinemia) in the fasting state in DM1 patients, but the percentage of DM1 patients reported to be affected by this was highly variable (Table 1). Increased plasma insulin was not accompanied by changes in the levels of fasting glucose. Hypoglycemia does not seem to occur in DM1 patients despite high levels of insulin. In response to glucose loading during glucose tolerance tests (GTT), the number of patients with impaired glucose tolerance curves was highly variable across studies. These differences cannot be explained by differences in the amount of glucose given during these tests. In patients, glucose loading was often accompanied by an exaggerated insulin response in comparison to controls. Although across studies, a significant number of patients had some form of impaired glucose tolerance, only a small minority of patients had GTT that met the criteria for diabetes mellitus. Insulin resistance can be defined as the inability of a known quantity of exogenous or endogenous insulin to increase glucose uptake and utilization in an individual as much as it does in a normal population (88). Perhaps the best-known example of another disease in which insulin resistance acts as one of the key initiating pathophysiological mechanisms, is type 2 diabetes mellitus. In type 2 diabetes mellitus, insulin resistance leads to disturbed glucose homeostasis, as reflected by the diagnostic criteria (89). The literature on insulin resistance in DM1 is contradictory. Evaluation was performed in many studies, using techniques like calculation of the homeostatic model assessment for insulin resistance (i.e., HOMA-IR), insulin tolerance testing and euglycemic insulin (i.e., glucose clamp) infusion (Table 1). Results varied from strong evidence for insulin resistance in a majority of patients to results comparable to controls. Seemingly discrepant results across studies could relate to specific patient populations and underpowered sample sizes, selection of controls, frequency of sampling during tolerance tests, and controlling for confounders, such as lean body mass. Also, the use of a double antibody radioimmunoassay in many of the older studies, may have overestimated insulin levels as a result of cross-reactivity with proinsulin, levels of which have been established to be elevated in DM1 (80, 84). In summary, a subset of (but not all) patients with DM1 present with hyperinsulinemia, which occurs during fasting, but also as an exaggerated (pro-)insulin response to carbohydrate intake. Possibly, the hyper(pro-)insulinemia in DM1 reflects an appropriate and partly successful compensatory change in response to insulin resistance that may occur in DM1. This is reflected by impaired glucose tolerance and/or abnormal glucose tolerance curves and increased levels of HbA1c, but a relatively infrequent occurrence of type 2 diabetes.

**Table 1:** Clinical insulin studies in myotonic dystrophy type 1.

**References**	**No. P**	**No. HC**	**No. DC**	**Insulin assay**	**Fast. insulin**	**Fast. glucose**	**Glucose tolerance test (GTT)**	**[Insulin] during GTT**	**No. (%) impaired GTT**	**No. DM**	**Glucose disposal during EII**	**Comments/Results**
Marshall et al. (56)	11	0	0						2 (18%)	0		ITT demonstrates mild insulin insensitivity in 1 patient
Huff et al. (57)	6	14	6	Double antibody RIA	↑	N	100 g	↑	5 (83%)			ITT demonstrates normal response in 4/6 patients. Normal insulin clearance
Huff and Lebovitz (58)	8	0	6		↑	N						
Bundey (59)	11		3	Double antibody RIA	N					0		
Goden et al. (60)	12	6	0		↑	N	100 g	↑	0 (0%)	0		Normal glucose tolerance curve. ITT apparently normal
Jackson et al. (61)	3	0	0	Double antibody RIA	↑	N	50 g	N to ↑	0 (0%)	0		
Mendelsohn et al. (62)	11	45	0		N to ↑	N	1.75 g/kg	N to ↑ [1]	3 (27%)	0		
Walsh et al. (63)	20		2	double antibody RIA	↑	N	100 g	↑	2 (10%)	1		Excessive plasma insulin response
Bird and Tzagournis (64)	10	233	165			N		N to ↑	4 (40%)	0		Normal response to exogenous insulin
Bjorntorp et al. (65)	17	35	0		N to ↑	N	100 g	N		0		
Barbosa et al. (66)	29	30	0	Immuno-assay	↑		100 g	↑	13 (38%)	2		Abnormal glucose tolerance curve. ITT shows decreased insulin sensitivity in DM1 patients
Nuttall et al. (67)	12	18	0		↑	N	100 g	↑		0		ITT demonstrates no differences in patients vs. controls
Poffenbarger et al. (68)	8	8	0		N	↑	100 g	↑		0		Increased proinsulin secretion in parallel with insulin hypersecretion
Kobayashi et al. (69)	7	7	0		N	N	1.75 g/kg	↑		0		Abnormal glucose tolerance curve
Tevaarwerk and Hudson (70)	14	25	5	Radioligand method	↑		50 g	↑	12 (86%)			Abnormal glucose tolerance curve. ITT demonstrates insulin insensitivity in 14/14 patients
Moxley et al. (71)	6	7	6	Double antibody technique	N	N	1.5 g/kg	↑	0 (0%)	0		Decreased forearm muscle sensitivity to exogenous insulin in patients vs. healthy and neuromuscular disease controls
Festoff and Moore (72)	6	15	0		N	N	1.75 g/kg	N to ↑	2 (33%)	0		
Stuart et al. (73)	12				N to ↑ [3]			↑ [8]		0		ITT demonstrates insulin insensitivity in 9/12
Moxley et al. (74)	6	13	0		N	N		↑	0 (0%)	0	↓	Decreased glucose disposal, even when controlled for 24 h-creatinin clearance. Euglemic insulin infusions demonstrate normal insulin clearance
Corbett et al. (75)	3	4	3	Double antibody RIA	N	N				0	↓	Normal insulin clearance
Hudson et al. (76)	10	17	22	Double antibody RIA	↑	N						
Moxley et al. (77)	9	29	0	Double antibody technique	↑		15–25 g	↑	0 (0%)	0	↓	Normal glucose tolerance curves. No increase in insulin sensitivity after glucose loading in DM1 patients vs. controls, even after correction for differences in muscle mass
Krentz et al. (78)	10	10	0	Double antibody RIA	↑	N	75 g	↑	0	0		Abnormal glucose tolerance curves in DM1 compared to controls, but no patient met criterium for impaired glucose intolerance
Piccardo et al. (79)								↑	0	0		Normal glucose tolerance. ITT: decreased insulin sensitivity in patients vs. controls
Krentz et al. (80)	10	10	0	Double antibody RIA; immunoradio-metric	N to ↑	N	75 g		0 (0%)	0		Fasting insulin results depending on assay used. Increased fasting prosinsulin. Normal fasting C-peptide
Gomez Saez et al. (81)	12	14	0	RIA	N to ↑ [3]		75 g	↑	2 (17%)	1 (8%)		C-peptide normal during fasting; increased in patients vs. controls during GTT
Annane et al. (82)	11	11	0			N	50 g	↑	N	0		
Johansson et al. (83)	18	18	0	Double antibody RIA	↑	N	75 g	↑	3 (17%)	Excl.		HOMA-IR 2.3 in patients vs. 1.4 in controls
Perseghin et al. (84)	10	10	8	Microparticle enzyme immunoassay	N	N	75 g	N		Excl.	N	Mildly abnormal glucose tolerance curve. Increased proinsulin in fasting state and during EIC testing. Normal fasting C-peptide. Decreased lean body mass in DM1
Perseghin et al. (85)	10	10	0	Microparticle enzyme immunoassay		N				Excl.	N	No alterations in carbohydrate or lipid metabolism in patients vs. controls when controlled for lean body mass; abnormal regulation of protein breakdown in DM1. Lower IGF-1 levels in DM1
Matsumura et al. (86)	95	734	0		↑	N	75 g	↑	14 (15%)	9		HOMA-IR 1.96 in patients vs. 1.36 in controls; positive correlation between insulinogenic index and insulin resistance. ITT demonstrates impaired whole body glucose tolerance
Rakocevic Stojanovic et al. (87)	34	34	0	RIA	↑	N	75 g		0	0		HOMA-IR 4.2 in patients

*Empty cells denote information not present in the study or not available to us; N, normal or not significantly different from controls; ↓↑ denote decreased or increased values in comparison with controls, respectively. DM, diabetes mellitus; EII, euglycemic insulin infusion; Fast., fasting; GTT, oral glucose tolerance test; HOMA-IR index, homeostasis model assessment-insulin resistance index; ITT, insulin tolerance test; No., number; RIA, radioimmunoassay; No., number of patients [P], healthy controls [HC], or disease controls [DC]*.

### Relevance of Insulin Resistance

**Figure 2:**
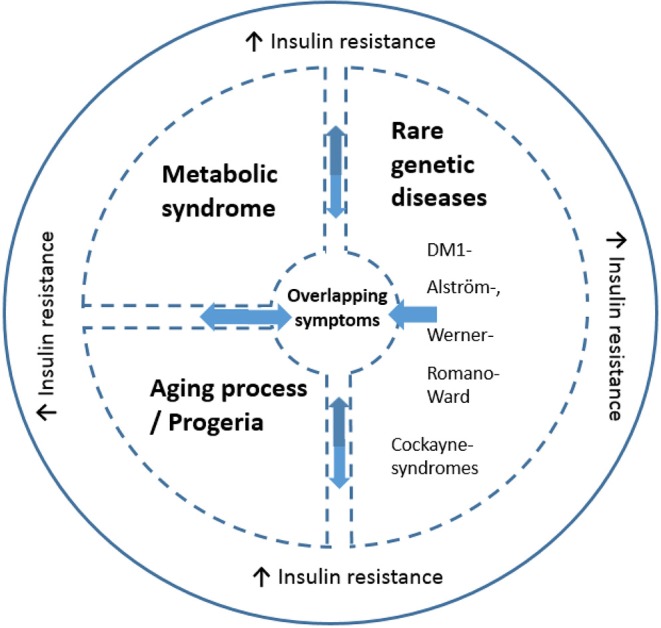
Schematic illustration of the link between altered insulin resistance/signaling and common symptoms across disorders (including DM1). The message is that some DM1 related symptoms are found in Metabolic syndrome, progeria and the rare genetic disease, while insulin resistance is an overlapping symptom seen all diseases. The common symptoms include but are not necessarily present in all overlapping disorders: cataracts, skull hyperostosis, early hair graying/loss, impaired muscular development or wasting, cardiac arrhythmia, hypogonadism, testicular atrophy and dyslipidemia. Insulin resistance is a key feature in some but not all DM1 cases and is present in 5–17% of DM1 cases. DM1 has symptomatic (and insulin resistance) overlap with certain rare diseases, including Alström-, Werner-, Romano-Ward, Cockayne syndromes, and ataxia telangiectasia. Both normal aging and DM1 are associated with increased insulin resistance. Moreover, DM1 is conceptualized as a disease with early-onset aging. Metabolic syndrome is also linked to insulin resistance (90), and is present in DM1 (87, 91).

The presence of insulin resistance in DM1 is of interest for several reasons. First, insulin resistance is one of the most important features of metabolic syndrome (90). The prevalence of metabolic syndrome in DM1 has been estimated at 17% (87, 91). Several of its features, such as hypertriglyceridemia (67%) and low HDL cholesterol (35%) occur with high frequency in DM1, whereas other features, such as hypertension, are relatively infrequent in DM1 (91). Secondly, besides type 2 diabetes mellitus and myotonic dystrophy, insulin resistance is a feature of several rare genetic diseases: Alström, Werner, Cockayne, Romano-Ward syndromes, and ataxia telangiectasia. Interestingly, many clinical characteristics of these diseases overlap with those of DM1: notably cataracts, early graying and loss of hair, impaired muscular development or wasting, skull hyperostosis, hypogonadism and testicular atrophy, and dyslipidemia (76). While these rare diseases have distinct pathologies and mechanisms from DM1, they also share a number of symptoms and insulin resistance with DM1. These are highlighted in Figure 2. Alström syndrome is a rare autosomal recessive genetic disorder characterized by truncal obesity in juveniles, insulin resistance and hyperinsulinemia, type 2 diabetes, hypertriglyceridemia, decreased stature in adulthood, cone-rod dystrophy, deafness, cardiomyopathy, and progressive pulmonary, hepatic, and renal dysfunction (92). Werner-syndrome (WS) is a prototypical segmental progeroid syndrome characterized by multiple features consistent with accelerated aging. Early stage WS is associated with skin atrophy, loss of subcutaneous fat and hair thinning/loss. Bilateral cataracts appear during middle age in association with type 2 diabetes, hypogonadism, osteoporosis, atherosclerosis and malignancies, and gonadal atrophy (93). Romano-Ward syndrome is an autosomal dominant genetic condition associated with mutations in the KCNQ1, KCNH2, and SCN5A genes. Clinically, a prolonged cardiac ECG QT interval, insulin resistance and deafness are observed in addition to cardiac arrhythmia which can lead to heart failure (94). Cockayne syndrome is a rare and fatal autosomal recessive neurodegenerative disorder characterized by growth failure, severe neurological manifestations including sensorineural deafness, cognitive deficits, pigmentary retinopathy, cataracts, and ambulatory and feeding difficulties (95). Ataxia telangiectasia is a rare, neurodegenerative, autosomal recessive disease causing severe disability with a broad spectrum of clinical features, including progressive cerebellar ataxia, oculocutaneous telangiectasia, variable immunodeficiency, susceptibility to malignancies, and increased metabolic diseases. Mutations in the ATM ataxia-telangiectasia gene are responsible for the functional impairments in angiogenesis regulation and glucose metabolism (96). Whether insulin resistance contributes to the commonality in symptoms of these rare diseases, metabolic syndrome and progreria with DM1 is unestablished. In the case of DM2, insulin resistance prevalence in DM2 is far less compared to in DM1 (12) suggesting that mechanisms including insulin resistance may differentiate between DM1 and DM2 phenotypes.

**Figure 3:**
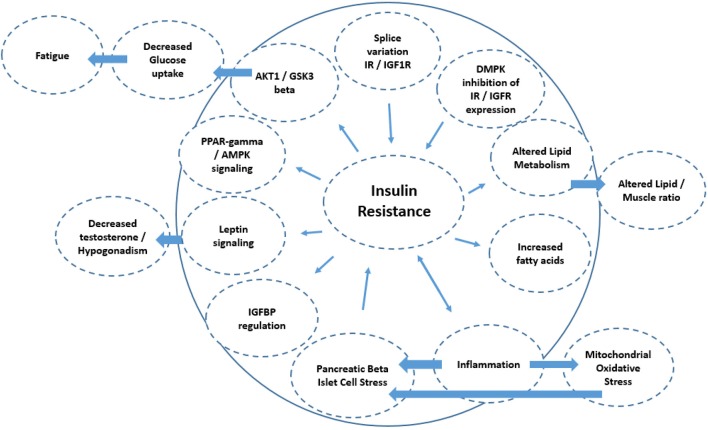
Schematic diagram of the mechanisms associated with insulin resistance. The directionality of the arrow indicates if it is a cause or consequence of insulin resistance. This includes splice variation of IR/IGF1 receptor expression (e.g., IR-A isoform increase vs. IR-B), leading to insulin resistance. Diminished cell membrane IGFR and IR expression could result from the DMPK repeat length expansions blocking the nuclear pores, and also result in insulin resistance. Alterations in lipid metabolism as a consequence of insulin resistance may also lead to altered lipid/skeletal muscle ratios, as is seen in DM1. Reduced insulin sensitivity is also associated with diminished muscle protein synthesis, resulting in decreased muscle mass. Inflammation has a bidirectional relationship with insulin resistance and may in part underlie atherosclerosis in DM1. Mechanistically, inflammation increases mitochondrial oxidative stress and stress at the level of the pancreatic beta islet cells which also increases insulin resistance. Decreased IGFBP expression also reduces insulin sensitivity by reducing the half-life of any insulin-IGFBP complex to bind to the IR/IGF receptors. Insulin resistance also acts to alter leptin signaling which amongst others can regulate testosterone tone. Insulin resistance can also act at the level of (1) PPARγ/AMPK signaling and (2) AKT1/GSK3β signaling thereby regulating glucose turnover which may be linked to fatigue in DM1.

These effects of insulin in the context of DM1 are summarized in Figure 2 with putative mechanisms interacting with insulin resistance relevant to DM1 summarized in Figure 3. Moreover, these clinical observations raise the question what role insulin resistance plays in the development of certain phenotypes common to these diseases. A common denominator to some of these diseases may be an accelerated aging process/progeria. Indeed, DM1 has been suggested to be a progeroid disease (97–99). Furthermore, increasing insulin resistance with age is noted in the general population (100). Part of the clinical spectrum of progeroid diseases is an increased risk of malignancy. DM1 is associated with an increased risk of certain cancers, for example, thyroid cancer (101, 102). Of note, hyperinsulinemia is also associated with an increased risk of thyroid cancer [reviewed in (23)]. Given the diversity of somatic symptoms in DM1, the impact of insulin resistance affects a range of tissues including skeletal muscle, cardiovascular and adipose tissues. For instance, skeletal muscle is highly dependent on glucose transport with ~75% of all plasma glucose estimated to be transferred into skeletal muscle to initiate and maintain movement. Since increased insulin resistance results in impairment to glucose transport, notably via the GLUT4 transporter, this has a profound impact on muscle function and is associated with dysregulated mitochondrial function. Of note, skeletal muscles from type 2 diabetes and obesity patients have smaller mitochondria and impaired electron transport compared to healthy age-matched controls. Impaired mitochondria functionality through decreased functionality of oxidative enzymes have been shown to result in fatty acid accumulation in muscle, insulin resistance, type 2 diabetes and obesity (103). Changes in lifestyle and diet could lead to differences in mitochondrial morphology with impairment or loss of mitochondrial function (104). In DM1, irregular cristae in the mitochondria of red muscle fibers, i.e., type I muscle fibers, has been reported (105), which was associated with fatigue. Although interpretation of this research is difficult, it is not known if this was also associated with increased insulin resistance (which has been independently liked to aberrant cristae morphology).

Insulin resistance also has consequences for cardiovascular symptoms notably cardiac failure in DM1. Left ventricular hypertrophy is amongst others caused by insulin resistance. Arterial stiffness, results from insulin resistance induced lipid metabolism alterations. This results in a loss of arterial elasticity, with a difference in blood pressure and higher pulse-wave velocity and augmentation (106). Untreated arterial stiffness can result in carotid intima-media thickness leading to carotid atherosclerosis, with higher risk for cardiovascular diseases (106).

It is well-established that those with insulin resistance have an increased prevalence of type 2 diabetes, and a 2–3 times higher risk for cardiovascular disease (107–110). Heart failure associated with type 2 diabetes results from impaired microvascular functioning and myocardial perfusion dysfunction (108). The subsequent cardiac hypertrophy leads to chronic cardiac stress and finally to heart failure (111). Insulin resistance can also alter systemic lipid metabolism which then leads to the development of dyslipidemia and the well-known lipid triad: (1) high levels of plasma triglycerides, (2) low levels of high-density lipoprotein, and (3) the appearance of small dense low-density lipoproteins which impact on cardiovascular function. This triad, along with endothelial dysfunction, which result from aberrant insulin signaling, contribute to atherosclerotic plaque formation. Regarding the systemic consequences associated with insulin resistance and the metabolic cardiac alterations, it can be concluded that insulin resistance in the myocardium generates damage by at least three different mechanisms: (1) signal transduction alteration, (2) impaired regulation of substrate metabolism, and (3) altered delivery of substrates to the myocardium. In DM1 there is a concurrent high level of plasma triglycerides, increased body fat mass and increased insulin resistance. However, type 2 diabetes is unusual, and blood pressure is not elevated in DM1 patients (112), but atherosclerosis and heart rhythm disturbance can be present in DM1 (113).

Insulin resistance is associated with dyslipidemia both in terms of lipogenesis and lipolysis. The anti-lipolytic effect of insulin requires lower insulin concentrations than that required for the activation of glucose transport processes. As a consequence, impairment of glucose transport resulting from insulin resistance does not necessarily result in parallel dysregulation to insulin's anti-lipolytic effect. As a result adipose stores may remain unaffected or even increase in the face of increased insulin resistance. In DM1, lipid metabolism is dysregulated, particularly in skeletal tissues (114). Adipose tissue acts as an energy depot for skeletal muscles. The secretion of adipokines by adipocytes includes the regulation of mitochondria present in adipose tissues and plays a role in regulating the lipid metabolism and glucose metabolism in the brain and peripheral organs (115). Similar to adipokine secretion by adipose tissues, muscles secrete myokines which control muscle to fat ratio. This proportion of muscle to fat influences body composition, shape and metabolic homeostasis (116). In DM1, the fat mass index (FMI) is lower compared to healthy controls. As FMI increases, this is associated with hypertriglyceridemia. Furthermore, total FMI increases are reported to significantly correlate with increased muscular disability rating and decreased motor and lung functionality (117). Taken together this may suggest that insulin resistance could play a role in fat regulation which impacts on DM1 symptoms.

### Brain Insulin Signaling: Relevant in DM1?

In addition to the alterations in insulin signaling and glucose homeostasis in peripheral tissues, brain glucose uptake has been shown to be decreased in DM1 (82, 118). It is currently unknown whether altered insulin signaling is involved, as cerebral glucose uptake is thought to be independent of insulin (17). Irrespective of its role in cerebral glucose uptake, insulin has various important functions in the brain, such as regulating synaptic plasticity [reviewed in (17, 47, 119)]. These actions may be summarized as those influencing food intake/goal-directed behavior, peripheral metabolism and cognition. As in peripheral tissues, insulin resistance, the reduced ability of insulin to exert its actions on target tissues, may develop in the brain. Brain insulin resistance in DM1 is currently an unexplored field, and direct evidence for altered brain insulin signaling in DM1 is lacking. However, we think its presence is not unlikely. It may result from pathophysiological mechanisms directly related to the CTG repeat expansion, but it could also be secondary to being overweight, which occurs in ~50% of patients (120). Insulin resistance in the brain may also alter the peripheral effects brain insulin has on metabolism (119). It could lead to increased peripheral insulin resistance, increased lipolysis exacerbating dyslipidaemia and increased body weight, all of which are common in DM1.

As brain phenotypes in DM1 are heterogeneous and many clinical features are part of the spectrum, some of them could be related to altered brain insulin signaling in DM1. For example, apathy is considered a hallmark symptom of DM1 (121). In an animal model, induced loss of function of insulin receptor signaling leads to a state of reduced motivation, thought to be translatable to a condition of apathy in humans (122). In addition, brain insulin resistance may lead to cognitive deficits, especially visuospatial and verbal memory deficits (119). These cognitive deficits are also observed in DM1 (123). Brain insulin signaling is also linked to affective state, disturbances of which may be involved in depressive symptoms observed in DM1 (124, 125). Obsessive-compulsive disorder (OCD) personality traits are also a feature of DM1 (125). We have established that in OCD cohorts, alterations in insulin signaling occur (126), and that increased insulin resistance is associated with decreased behavioral flexibility (127). Underlying potential relations with clinical CNS features of DM1, insulin signaling could also play a role in the development of brain imaging alterations that have been reported in DM1. White matter hyperintensities (WMH) in periventricular and subcortical white matter in excess of those in matched controls, as well as microstructural alterations in all major projection, commissural and association fibers have been observed in DM1 (128, 129). In a recent study in cognitively normal middle-aged healthy individuals, hyperinsulinaemia was identified as an independent predictor of WMH (130). Insulin signaling may be involved in axonal guidance during development but also plays a key role in lipid metabolism, including that influencing myelination. Structural white matter networks in DM1 are different from controls, and this could represent a developmental or degenerative process, or both (131, 132). The role of insulin in synaptic homeostasis also raises the questions whether gray matter volume reductions in DM1, as observed in morphometry studies, are related to changes in insulin signaling.

### IGFs in DM1

In contrast to the many studies dedicated to the role of insulin and insulin resistance in DM1, the study of insulin-like growth factors and their signaling pathways has received less attention in DM1. Circulating IGF1 levels in DM1 patients are low in comparison with controls, which apply also for the vitamin D levels in these patients (85, 133). In view of DM1 as a progeria disease, it is of interest that the growth hormone/IGF1 system in combination with available serum vitamin D levels is implicated in the regulation of lifespan (99, 134, 135).

### Evidence From Clinical Trials

Besides cross-sectional clinical evidence for the involvement of insulin signaling in the development of DM1 phenotypes, the results of several therapeutic trials support its involvement. A recent trial of the anti-diabetic drug metformin in 40 adult DM1 patients showed promising effects on exercise capacity and mobility that warrant replication (136). Although the relative contribution of various working mechanisms of metformin to these effects remain speculative at this time, it is tentative to assume interference with insulin signaling in muscle cells, such as modulation of AMPK and PPARγ signaling as well as alteration in IGF1R splice variant expression may be involved (137). The utility of metformin for the management of hyperglycaemia in DM1 has already been reported (138). In another randomized double-blind study of long-term recombinant IGF1 administration in DM1 patients, beneficial effects on insulin sensitivity, glucose utilization, body composition and muscle function were observed (139). However, although similar beneficial effects on metabolic variables were observed, beneficial effect on muscle strength and function were not confirmed in a more recent, larger open-label clinical trial (25). The latter study evaluated recombinant IGF1 complexed with recombinant IGFBP-3. The authors concluded that longer, parallel group trials are indicated to further clarify the safety and efficacy of this therapy (25). Possibly, the addition of ALS glycoprotein (see above) to IGF1-IGFBP-3 could further improve the action of IGF1 in a future trial.

In summary, there is clinical evidence of alterations in insulin signaling in DM1, mainly based on studies focusing on carbohydrate metabolism. Alterations in lipid and protein metabolism in DM1 may also relate to alterations in insulin signaling but this has not been evaluated fully. For muscle phenotypes, clinical studies have hinted at the involvement of insulin signaling in muscle wasting by demonstrating favorable yet preliminary effects on muscle with recombinant IGF1 or metformin in DM1 patients. A role for insulin signaling in CNS involvement is plausible but currently remains unproven. Likewise, most relationships between alterations in insulin signaling and DM1 clinical features remain to be investigated. Clearly, further exploration of insulin-related signaling in relations to muscle and brain involvement in DM1 may represent a useful strategy in terms of disease-insight and therapeutic utility. In particular, studies have evaluated and demonstrated the feasibility, safety and efficacy of training/exercise in DM1 (140–142). The mechanisms through which exercise exerts its positive effects are unknown, but changes in insulin resistance could be a potential mechanism worth exploring in future studies.

## Molecular Evidence of Insulin Signaling Involvement in DM1

Decades after clinical alterations in insulin signaling had been observed, advances in pathophysiological insights have begun to shed light on the molecular correlates of these changes (summary of several representative studies shown in Table 2). In 2001, Savkur et al. demonstrated a relative increase in IR-A compared to IR-B isoform expression (see above) in muscle cells biopsied from patients with adult-onset DM1 (52). Experiments ruled out muscle cell regeneration or degeneration as an explanation, and indicated abnormal splicing regulation resulting from increased expression of the splicing regulator CUG-BP as a plausible explanation for a switch toward expression of the IR-A isoform. This switch toward IR-A was recaptured in muscle cultures from fibroblasts, which also demonstrated decreased insulin mediated glucose uptake (52). Dysregulated splicing of the INSR pre-mRNA toward the embyronal IR-A isoform in muscle cells was confirmed in subsequent studies (150, 152, 153). Splicing dysregulation of IR pre-mRNA was independent of fiber type (i.e., type 1 or type 2 muscle fibers), and of muscle location (i.e., proximal vs. distal) (150, 152). Moreover, IR expression at the protein level occurs only in type 1 muscle cells (153). This recent study by Renna et al. also demonstrated that in DM1 muscle cells, insulin-dependent activation of signaling pathways IRS1-AKT/PKB and Ras-ERK was decreased in comparison to controls (153). The results of this study suggest a link between dysregulated IR splicing and previous findings of decreased insulin mediated glucose uptake and protein synthesis in myotubes differentiated from fetal DM1 muscle myoblasts in comparison to healthy control myoblasts (148). However, the same group previously demonstrated defective post-receptor insulin signaling in human DM1 myotubes, despite similar IR-A:IR-B expression in comparison to control myotubes (152). Therefore, a post-receptor defect could contribute to insulin resistance in DM1, regardless of dysregulated IR splicing (152). Of note, treatment of differentiated DM1 myotubes with recombinant human IGF1 (rhIGF-1) led to partial recovery of glucose uptake and protein synthesis, providing a rationale for the clinical trials using rhIGF-1 (see above) (148).

**Table 2:** Molecular insulin studies in myotonic dystrophy type 1.

**References**	**No. P**	**No. HC**	**No. DC**	**Cell/tissue type**	**Glucose transport/oxidation**	**Insulin binding study**	**IR splicing analysis**	**Comments/Results**
Kobayashi et al. (69)	7	7		Monocytes	0	1	0	Similar insulin binding of monocytes in DM1 patients vs. controls
Tevaarwerk et al. (143)	12	12		Monocytes	0	1	0	Decreased insulin binding of DM1 monocytes compared to control monocytes
Festoff et al. (72)	6	15	0	Monocytes	0	1	0	Decreased insulin binding of monocytes in DM1 patients vs. controls
Moxley et al. (144)	9	10	2	Monocytes	0	1	0	Absence of post-prandial increase in monocyte insulin binding affinity in DM1 patients vs. controls
Mably et al. (145)	14	28	0	Adipose tissue	1	0	0	Decreased glucose transport in basal conditions; increased glucose transport and oxidation in insulin stimulated conditions
Hudson et al. (76)	10	0	22	Fibroblasts from skin biopsies	0	1	0	Decrease insulin binding of cultured fibroblasts in DM1 patients vs. controls, possibly as a result of decreased receptor numbers
Lam et al. (146)	10	10	0	Fibroblasts from skin biopsies	0	1	0	Decreased insulin binding in DM1 in comparison to controls
Morrone et al. (147)	9	4	6	Human DM1 skeletal muscle	0	0	0	Decreased IR mRNA in patients vs. controls. Reduction in IR protein
Furling et al. (148)	1		0	Human DM1 muscle cells purified from fetal myoblasts	1	1	0	Normal basal glucose uptake, but decreased glucose uptake after insulin stimulation. Decreased protein synthesis. Decreased IR mRNA expression and decreased insulin binding
Savkur et al. (52)				Human DM1 skeletal muscle	1	0	1	Aberrant regulation of insulin receptor alternative splicing is associated with insulin resistance in myotonic dystrophy
Guiraud-Dogan et al. (149)	n/a	n/a	n/a	Transgenic DM1 mouse tissues	1	0	1	DM1 CTG expansions affect insulin receptor isoforms expression in DM1 mouse models
Santoro et al. (150)	3		2	Human DM1 skeletal muscle	0	0	1	Alternative splicing of human insulin receptor gene (INSR) in type I and type II skeletal muscle fibers
Takarada et al. (151)				Fibroblasts; cell cultures	0	0	1	Resveratrol modulates IR splicing
Renna et al. (152)	8	8		Human DM1 skeletal muscle; myotubes derived from human DM1 myoblasts	1	0	1	Skeletal muscle biopsies: splicing dysregulation toward IR-A in patients. No relation between IR-A/IR-B expression ratio and HOMA-IR in patients. Altered insulin signaling pathways in patients vs. controls in terms of protein level and phosphorylation status, dependent upon which muscle was evaluated. Myotubes: Changes in insulin signaling pathways in DM1 vs. controls after insulin stimulation, despite similar IR-A/IR-B expression ratios across groups.
Renna et al. (153)	8	6		Human DM1 skeletal muscle	1	0	1	Aberrant insulin receptor expression is associated with insulin resistance and skeletal muscle atrophy

*Empty cells denote information not present in the study or not available to us. 0/1 indicates whether or not a certain analysis was carried out. DM1, myotonic dystrophy type 1; mRNA, messenger RNA; No., number of patients [P], healthy controls [HC], or disease controls [DC]; IR, insulin receptor*.

Besides dysregulated splicing toward embryonal isoforms of IR-A, studies evaluated, *ex vivo*, the expression of the IR in human DM1 muscle cells in comparison with healthy or neuromuscular disease controls. In human DM1 muscle cells derived from muscle biopsies from adult-onset DM1 patients, Morrone et al. showed decreased IR mRNA and protein in a well-controlled study (147). In line with this study, Furling et al. demonstrated decreased IR mRNA in myotubes derived from fetal DM1 myoblasts in comparison with “normal” myotubes. At the protein level, normal and diminished IR expression have been reported, with differences possibly related to whether analyses were corrected for fiber type distribution (52, 153). Previously, some, but not all, insulin binding studies in blood cells had demonstrated decreased insulin binding in DM1 cells compared to control cells, and authors have suggested decreased receptor numbers (72, 76) and/or decreased receptor affinity (143, 146) as potential explanations (Table 2). While decreased IR numbers are compatible with IR mRNA and protein expression level studies, decreased affinity would not, as the affinity of IR-A is in fact higher than that of IR-B (23). Furthermore, DM1-related changes in insulin signaling may be tissue specific.

In addition to the spliceopathy related alterations in the insulin receptor isoforms, haploinsufficiency of the normal *DMPK* gene product may lead to deficits in insulin signaling. It was demonstrated that *DMPK* knockout mice show impaired insulin signaling in tissue that normally express the *DMPK* protein (51). Clinically, these mice displayed metabolic derangements, such as abnormal glucose tolerance and reduced glucose uptake, possibly resulting from defective intracellular trafficking of insulin and IGF1 receptors (51). On the other hand, *DMPK* repeat expansion also influence insulin signaling in DM1. The expression of the homeobox gene Six5 is suppressed in DM1 (see section on DM1 pathophysiology, and Figure 3 above) (4). The level of expression of the IGF binding protein IGFBP5, is related to the degree of Six5 gene expression mouse models, thus providing another link between upstream pathophysiological processes and insulin signaling alterations in DM1 (4).

In summary, potential molecular mechanism that may help understand insulin resistance include dysregulated splicing toward an embryonal isoform of the IR (i.e., IR-A) and decreased expression of IR in muscle cells. However, deficits in insulin signaling beyond the IR could contribute to insulin resistance. One possibility is that DMPK over-expression results in inhibition of IR/IGF1R expression. As discussed earlier, DMPK is a member of the Rho-family of kinases which are associated with the intracellular trafficking of proteins including amongst others the insulin- and IGF1 receptors (51). Decreased insulin and IGF1 receptor expression may be related to the extent of the CUG repeat length expansion as a result of DMPK haploinsufficiency. This could potentially block the passage of nuclear produced mRNA across the nuclear membrane into the cytoplasm for trafficking and expression at the cell membrane (154). This in turn would lead to less available insulin and IGF1 receptor expression, with insulin resistance resulting (51).

In addition, there are potential mechanisms involving the generation of reactive oxygen species (ROS) and mitochondrial dysfunction in DM1 that may be relevant to changes in insulin signaling. Mitochondria are subcellular organelles able to provide ATP, synthesized by ADP and pyruvate, this energy is used for muscle cells through oxidative phosphorylation (155). Besides the bioenergetics function mitochondria have a key function in cell signaling, cell proliferation/death, aging, involvement in disease, metabolic events, maintaining cytosolic Ca^2+^ homeostasis, and reactive oxygen species (ROS) synthesis (156). ROS synthesis are key players for the onset of pathways in the initiation of mitochondrial dysfunction resulting in the pathogenesis of the atrophy and diminished efficacy of skeletal muscle (157) and eventually into diabetic impairments and insulin resistance (158). One of the functions of DMPK is that it exhibits a mitochondrial-related protective role against apoptosis through oxidative stress. Several studies have demonstrated that alterations in DMPK expression affect skeletal muscle related mitochondrial functionality (7, 159).

Inflammatory processes in DM1 may also play a role in alterations in insulin signaling (Figure 3). Pro-inflammatory cytokines, including interleukins 6 and 8 (IL-6 and IL-8) and tumor necrosis factor-α (TNFα), are key players in the onset of insulin resistance (160). TNFα is secreted by adipocytes and macrophages and reduces insulin transduction which could result in glucose metabolic disorders, insulin resistance and obesity possibly followed by the later stadium type 2 diabetes (116, 161). This is performed through the activation of the TNF receptor 1, resulting in diminished AMPK (AMP-activated protein kinase) activation, leading to diminished ACC (acetyl-CoA carboxylase) phosphorylation and fatty acid oxidation. This cascade results in the accumulation of diacylglycerol in the skeletal muscle with as consequence insulin resistance developing (116).

Another putative mechanism linking insulin resistance in DM1 could involve leptin signaling. Leptin production is regulated through overfeeding, hyperinsulinemia, glucocorticoids, and TNFα, while the decrease in leptin is associated by amongst others fasting, cAMP, testosterone and the growth hormone (GH). Increased leptin levels have been reported in DM1 patients compared to controls (87). This may be explained by insulin resistance, with increased insulin levels, increased cortisol levels resulting from alterations in glucose intake, higher TNFR-2 receptor expression, hypogonadism, and decreased testosterone levels reported in DM1. Testosterone is able to decrease leptin levels (87) and also provides a mechanistic explanation for alterations in insulin resistance via changes in testosterone/leptin signaling. Although other pathophysiological mechanisms could be involved in the development of insulin resistance in DM1, toxic aberrant mRNA resulting in dysregulated splicing of the insulin receptor mRNA is currently best supported in the literature. Of note, dysregulated IR splicing in muscle could provide a therapeutic target in DM1, as has been hinted at in a recent study (151).

Little is known about the role of insulin signaling and its potential alterations in DM1 in the brain. Changes in brain insulin signaling are however not inconceivable, given the fact that IR isoform expression was abnormally regulated in various insulin responsive tissues (like skeletal muscles, adipose tissue, liver and pancreas). However, in the brain of DM300-328 mice, the hypothalamus/thalamus tissue, the food intake related control center with the highest degree of insulin receptor expression, did not show difference in IRA isoform levels (149). At the histopathological level, brain involvement in DM1 could be classified as a tauopathy (162). A recent review has pointed out the intricate interrelations between tau and insulin signaling (163). Tau is linked to brain and peripheral insulin resistance and beta cell dysfunction; conversely, insulin resistance could also lead to tau dysfunction (163).

With regards to a role for IGF1 in DM1, the current literature is scarce from a molecular perspective. Its role in skeletal muscular growth has led to IGF1 being considered as a treatment for muscular frailty in DM1 (see previous section) (139). Reduced muscle mass and strength is seen in *mdx* mice (a model for Duchenne muscular dystrophy). Increasing expression of mIGF1 in this model improved muscle mass and strength, and lowered fibrosis in aged diaphragms (164). While this approach could also be deployed in DM1, this requires confirmation in both preclinical and clinical DM1 cohorts. Of interest, serum levels of IGF1 and IGFBP3 are reduced in the DMSXL mouse model of DM1 (165) but it is not known whether supplementing with IGF1 or increasing its expression would rescue the impairment in muscle function seen in this model.

## Summary and Future Directions

In this review, we have consolidated the clinical and molecular evidence for the involvement of insulin signaling in myotonic dystrophy type 1. Clear links have been established between pathophysiological concepts in DM1, such as spliceopathy and *DMPK* haploinsufficiency, and molecular alterations in insulin signaling. However, it is clear that from a clinical perspective, defective insulin signaling is not a constant feature in DM1. As with other DM1 phenotypes, insulin signaling abnormalities, such as insulin resistance, occur only in a subset of DM1 patients. Moreover, insulin resistance may be a contributor to the DM1 phenotype (and may act to exacerbate symptoms) but it is too early to speculate whether it is causative or merely associative. Given the presence of insulin resistance in a subset of DM1 patients, patient stratification will be important in future studies, especially clinical therapeutic trials as insulin-modifying therapies may be relevant to some DM1 patients but not all. With respect to the latter, several smaller clinical trials that have modulated insulin signaling (e.g., metformin, recombinant IGF1) have shown results that deserve further exploration in larger patient samples. Questions that remain to be answered include which clinical DM1 features are linked to disordered insulin signaling in DM1, as alterations in insulin signaling and its effects may be tissue dependent. The relevance of changes in insulin signaling may vary dependent upon the DM1 feature. Mapping of clinical signs/symptoms to insulin-related changes would be useful to ascertain. Furthermore, whether modification of insulin signaling with drugs is feasible and effective in ameliorating these symptoms remains to be studied further. The evaluation of different kinds of type 2 diabetes mellitus medication for clinical efficacy in DM1 would also be useful. Elucidating the underlying molecular mechanisms of any drugs (e.g., metformin) with a positive clinical impact in DM1 is also a priority. These investigations of the molecular aspects should also assess any differential signaling between various down-stream insulin signaling pathways in DM1 and relate these to drug efficacy. Moreover, the efficacy of IGF1/2 should be re-evaluated in light of improvements to its action when combined with IGFBPs and ALS. As an example, the IGFBP6-IGF2 complex is reported to regulate muscle differentiation (166), which may be relevant in light of muscle loss in DM1. Whether markers, such as the IGFBPs and ALS change in DM1 requires systematic investigation. The therapeutic opportunities are not limited to pharmacology. Given the positive outcome of the OPTIMISTIC clinical trial examining cognitive behavioral therapy and exercise in DM1, it is of interest to know if alterations in lifestyle in DM1 patients that may beneficially impact health status, affect insulin signaling in DM1, and by what mechanisms. In future trials, candidate drugs could be combined with lifestyle change directed interventions to maximize therapeutic potential. In conclusion, insulin signaling in DM1 poses interesting challenges and potential therapeutic opportunities in the next decades.

## Author Contributions

SN, KO, and JG produced the concept, evaluated the literature, drafted and critically revised the manuscript while JW, PB, PH, and BE critically edited the manuscript. All authors contributed to the finalization of the manuscript for submission.

### Conflict of Interest

JG in the past 4 years has been a consultant to Boehringer Ingelheim GmbH. He was not an employee of this company, and was not a stock shareholder of this company. The funding organizations listed have had no involvement with the conception, design, data analysis and interpretation, review and/or any other aspects relating to this paper. PB is employed by a commercial company, namely VDL Enabling Technologies Group B.V., Eindhoven, Netherlands. This relationship did not in any way influence this manuscript. The remaining authors declare that the research was conducted in the absence of any commercial or financial relationships that could be construed as a potential conflict of interest.
